# Systematic assessment of sequencing depth requirements for Hi-C-derived metrics

**DOI:** 10.1186/s12864-026-13455-7

**Published:** 2026-10-15

**Authors:** Alexander Granovsky, Kirill Polovnikov

**Affiliations:** 1 AI Center, Skolkovo Institute of Science and Technology, Moscow, 121205 Russian Federation; 2 Institute for Physics & Astronomy, University of Potsdam, 14476 Potsdam-Golm, Germany

**Keywords:** Hi-C, Sequencing depth, Chromatin organization, A/B compartments, Insulation score, Loop extrusion, Contact probability, Biological replicates, Benchmark

## Abstract

**Background:**

Hi-C experiments produce genome-wide chromatin contact maps from which structural features can be quantified across multiple genomic scales, ranging from megabase-scale compartments to kilobase-scale boundaries and chromatin loops. Although high-resolution analyses commonly rely on hundreds of millions of sequenced read pairs, the minimum sequencing depth required for different classes of Hi-C-derived features has not been systematically established. We therefore sought to determine these depth requirements systematically.

**Results:**

We performed progressive random subsampling of twelve Hi-C libraries representing multiple cell types and experimental protocols. Loop-density and loop-size inference from the *P(s)* log-derivative was evaluated across all libraries, whereas compartment, insulation, and boundary analyses were performed on a subset of nine libraries with comparable full-depth coverage. Rather than defining a “sufficient depth” as an arbitrary fraction of the full-depth value, we introduced a biologically motivated criterion based on the variability of the metric between independent biological replicates. The required sequencing depth for each metric was defined as the point at which the subsampling scatter first reached the variability observed between independent biological replicates, representing the accuracy that additional sequencing cannot improve upon. Using this criterion, the genome-average loop-density estimate reached its biological floor at approximately 7 million read pairs, the genome-average loop-size estimate at approximately 20 million, insulation scores at approximately 80 million, and compartment eigenvectors at 100-kilobase resolution at approximately 110 million read pairs.

**Conclusions:**

Different Hi-C-derived metrics require substantially different sequencing depths to achieve biologically meaningful accuracy. For all metrics, depth-induced variability fell below biological replicate variability well before full sequencing depth was reached. These thresholds apply to genome-averaged characteristics, not local metrics of specific loci. They therefore provide practical guidance for genome-wide experimental design and sequencing budget allocation, suggesting that increasing the number of biological replicates is likely to improve the inference of global biophysical features more effectively than sequencing individual libraries to greater depth.

**Supplementary Information:**

The online version contains supplementary material available at https://doi.org/10.1186/s12864-026-13455-7.

## Background

Chromosome conformation capture techniques, and Hi-C in particular, have become central tools for studying the three-dimensional organization of the genome [1, 2]. In a Hi-C experiment, chromatin contacts are converted into a genome-wide matrix in which each entry reports how often two genomic loci are found in spatial proximity. This matrix contains information across a wide range of genomic scales. At megabase scales, it reveals A/B compartments, which reflect the spatial segregation of transcriptionally active and inactive chromatin [1, 2]. At submegabase scales, it reveals domains and domain boundaries, often quantified through insulation profiles [3]. At still finer scales, local enrichments of contacts appear as dots or loops, many of which are associated with cohesin-mediated loop extrusion and convergent CTCF sites [1, 4]. Finally, even after averaging over genomic position, the contact probability *P(s)*, where *s* is genomic separation, carries information about the polymer state of chromosomes and about the statistical properties of cohesin-mediated loops [5, 6].

These different features are not extracted from Hi-C maps in the same way. Compartment annotations are usually inferred from eigenvectors of observed/expected contact matrices at relatively coarse resolution. Insulation profiles are local tracks computed from contact density around the diagonal [3] and are therefore more sensitive to the number of contacts in a local genomic window. Individual loops or dots are even more local: they correspond to enriched pixels or small pixel neighbourhoods and are among the most demanding Hi-C features to detect reproducibly. In contrast, the contact probability *P(s)* is a genome-wide average over all pairs of loci at the same genomic separation. Therefore, different Hi-C-derived metrics use different amounts of averaging and should not be expected to have the same sensitivity to the sequencing depth.

Sequencing depth is consequently a central experimental-design parameter. Modern Hi-C libraries are often sequenced to hundreds of millions of read pairs, and the deepest reference maps can contain billions of contacts [1, 7, 8]. Although the per-base cost has decreased, sequencing depth remains the main variable cost that scales with the desired number of read pairs. Library preparation introduces a substantial fixed per-sample cost, but deeper sequencing increases the cost of each library linearly. Thus, reducing sequencing depth can substantially lower the sequencing component and make larger designs with more conditions, perturbations or biological replicates more feasible. The practical question is therefore how much sequencing is required for the specific Hi-C-derived metric that a study aims to use.

Several previous studies have addressed related questions, but usually in more restricted settings. One line of work focused on reproducibility of whole contact maps. Methods such as HiCRep, HiC-spector and GenomeDISCO were developed to compare Hi-C maps while accounting for distance dependence, smoothing, spectral properties or diffusion on graphs [9–11]. A later systematic comparison showed that reproducibility scores are themselves coverage-dependent and that Hi-C datasets should be compared at matched coverage. For loop density and loop size, critical depth was defined as the point at which the mean subsampling CV crossed the corresponding full-depth biological-replicate CV floor [12]. For compartment eigenvectors and insulation profiles, critical depth was defined as the point at which the mean correlation with the full-depth track crossed the corresponding full-depth biological-replicate correlation floor. Central values were obtained by log-linear interpolation of the mean curve between the two analysed depths bracketing the floor; ranges show the spread between libraries (interpolation of the ±1 SD envelope), not confidence intervals. These studies were essential for quality control and replicate comparison, but their primary object was the reproducibility score, not the depth requirement of individual biophysical features.

A second line of work considered sequencing depth in the context of particular feature callers, especially loop or dot detection. Loop calling is intrinsically local, because a loop is identified as a focal enrichment over its local background. As a result, loop detection is highly sensitive to sequencing depth, resolution, protocol and statistical thresholding. In a systematic comparison of chromosome conformation capture protocols by Oksuz et al., sequencing depth had a strong effect on the number and strength of detected loops, and formaldehyde-only protocols were more depth-sensitive than protocols using dual FA + DSG cross-linking [7]. The same paper further emphasized that compartments can be resolved with tens of millions of valid pairs, whereas individual loop detection may require much deeper sequencing, often hundreds of millions to more than a billion reads depending on the target resolution and the protocol [7]. Loop-calling tools such as Mustache also used downsampling analyses to test robustness of called loops, but these analyses were necessarily framed around tool-specific loop recovery from very deep reference maps [13]. More recently, Parker et al. treated the problem as one of statistical power for differential loop detection and showed that detecting changes in individual loops can require extremely deep maps, with billions of contacts per condition needed for well-powered detection of two-fold loop changes in a large fraction of loops [14].

These studies establish an important point: individual loops or dots are local and demanding Hi-C features. Our study addresses a different question. We do not analyse how individual loop strengths or loop calls degrade upon downsampling. Instead, we focus on genome-wide average metrics of chromosome organization: the mean density and size (genomic length) of random cohesin-mediated loops, compartment eigenvectors and compartment strength, and genome-wide insulation and boundary tracks. In contrast to the individual loop calls, *P(s)*-derived loop density and loop size are genome-averaged readouts of loop extrusion, not measurements of a specific CTCF-stabilized loop (a dot) in a contact map. Because they average over many loci, these parameters may remain stable at much lower depth than individual loop calls.

A third limitation of previous depth analyses is the definition of “sufficient” depth. Many studies compare downsampled maps to a full-depth reference and report how a metric changes as a fraction of the full-depth value. This is useful, but it leaves open the question of what fraction is acceptable. Moreover, different metrics also have different natural levels of biological reproducibility, because each Hi-C library samples a finite subpopulation from a heterogeneous ensemble of chromatin conformations rather than a single deterministic genome-folding state. A metric that is nearly identical between independent biological replicates requires very small depth-induced error to be considered reliable. A metric that varies substantially between biological replicates may reach its acceptable accuracy limit at lower sequencing depth, because additional reads cannot remove the biological variability intrinsic to the measurement.

We therefore define a natural threshold for sequencing depth using the biological-replicate variability. For each metric, we ask when the scatter introduced by random subsampling becomes comparable to the variability observed between independent biological replicates of the same cell line at the full depth. This biological-replicate floor approximates the accuracy limit of the metric under the present experimental conditions: no amount of additional sequencing of one library can eliminate differences that arise between independently prepared cell populations. We also report the corresponding technical-replicate floor, estimated from independent re-sequencing runs of the same library, which represents a tighter reproducibility expected when biological and library-preparation variation are minimized. This framework turns the sequencing-depth choice from an arbitrary percentage-of-full-depth rule into a metric-specific comparison against the natural biological variability of chromatin folding.

Here we apply this framework to twelve publicly available human Hi-C libraries from two studies: eight deeply sequenced libraries from Oksuz et al., spanning two cell types and two cross-linking protocols, and four in situ Hi-C libraries from Rao et al. [1, 7]. We subsample raw FASTQ read pairs from the full depth down to 200 M, 100 M, 50 M, 25 M, 10 M and 1 M read pairs, and process each subsample through the same pipeline. We then quantify the stability of four classes of metrics: loop density inferred from the short-distance dip of the *P(s)* log-derivative [6], loop size inferred from the loop-size-associated peak [5], compartment eigenvectors, and insulation profiles. The resulting benchmark places these Hi-C-derived metrics on a common scale and provides practical depth recommendations intrinsically tied to biological reproducibility.

## Methods

### Data acquisition and processing

Twelve Hi-C libraries were obtained from SRA: eight from Oksuz et al. [7] and four from Rao et al. [1]. Raw reads (2 × 50 bp for Oksuz et al., 2 × 101 bp for Rao et al.) were processed with the distiller-nf v0.3.4 pipeline [15] (environment: bwa-mem2 2.3, samtools 1.16.1, pairtools 1.1.2, cooler 0.10.3), using the BWA-MEM2 algorithm [16] for alignment, pairtools [17] and cooler [18], to hg38, with mapq30 filtering and multi-resolution .mcool generation from 1 kb to 10 Mb. Contact matrices were balanced (cooler balance) with default mad-max bin filtering. Bins lacking balancing weights (low-coverage or low-mappability bins) carry NaN weights and are excluded from all per-bin correlations. Analyses were restricted to autosomes and chrX. ChrX was retained in all E1 and insulation correlations. Downstream analyses used cooltools 0.7.1 [19], cooler 0.10.3 [18], bioframe 0.8.0, pairtools 1.1.2, NumPy 1.26.4, pandas 2.3.3, SciPy 1.15.3 and matplotlib 3.10.7.

### Definition of sequencing depth

Throughout, “depth” refers to the number of raw sequenced read pairs – the quantity specified when ordering sequencing – not to mapped, valid, deduplicated, or cis contacts. Subsampling was applied to the raw FASTQ read pairs before alignment, so that each target depth reflects a real sequencing budget; the downstream funnel (mapping rate, valid-pair rate, cis fraction) scales approximately in proportion to raw depth because each subsample is processed independently through the identical pipeline. Metrics are therefore normalised by raw read pairs, and the full single library is the per-library reference.

### Subsampling

Full FASTQ files were subsampled to target depths of 200 M, 100 M, 50 M, 25 M, 10 M, and 1 M read pairs using rasusa 3.0.0 software [20]. For every library, 25, 20, 20, 10, 5 and 4 independent random subsamples were generated at 1 M, 10 M, 25 M, 50 M, 100 M and 200 M read pairs, respectively. The rasusa random seed was set to the index of the subsample within this design (1–4 at 200 M, 5–9 at 100 M, 10–19 at 50 M, 20–39 at 25 M, 40–59 at 10 M and 60–84 at 1 M), identical across libraries. Each subsample was independently processed through the full distiller-nf pipeline.

### Loop-density and loop-size inference

**Fig. 1:**
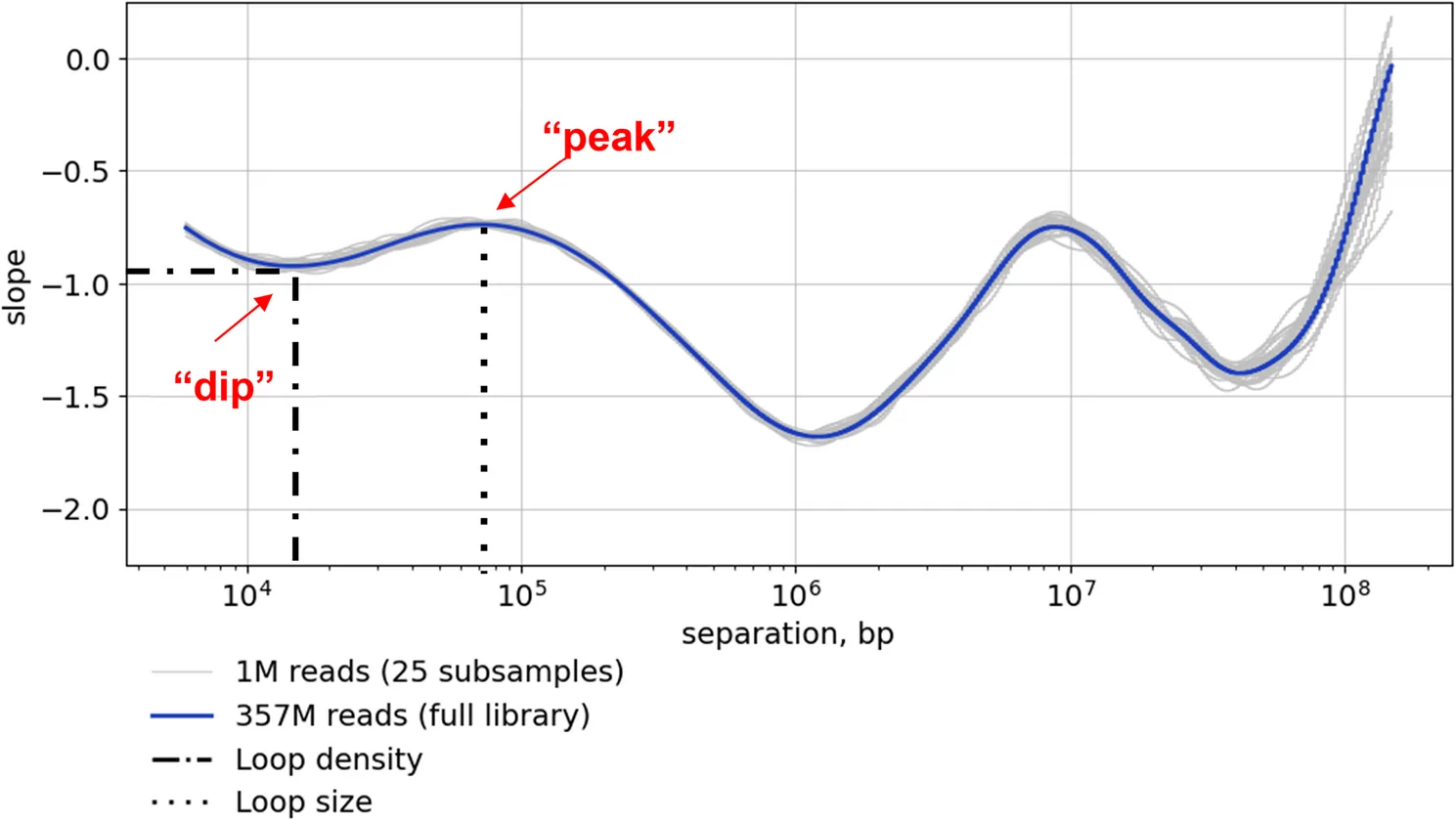
Robustness of P(s)-derived loop features to extreme downsampling. Example log-derivative of the contact probability, $\:dlogP\left(s\right)/dlog\:s$, as a function of genomic separation s for the hESC-FA-DpnII-R1-T1 Hi-C library. The full-depth library contains 357 million read pairs and is shown in blue; twenty-five of the independent 1M-read subsamples are shown in gray for visual clarity. The short-distance local minimum (“dip”, approximately 15 kb) is used to infer loop density [6], whereas the local maximum (“peak”, approximately 74 kb) is used for the estimate of the loop size [5]. Both features remain visually stable after approximately 350-fold downsampling, illustrating the robustness of genome-wide *P(s)*-based metrics

Contact probability *P(s)* was computed with cooltools.expected_cis [19] using smoothed (σ = 0.1), genome-aggregated cis expected values on balanced counts. The log-derivative was evaluated numerically from *P(s)*; the short-scale minimum (dip) and the loop-size maximum (peak) were both extracted (Fig. 1): the dip as the min of the slope within a 6–90 kb window, and the peak as the max within a 40–220 kb window. Both positions are resolved at the 1 kb bin size. The loop period T (inverse loop density) and effective fragment length $\:{{v}_{0}}^{eff}$ were inferred by fitting the dip coordinates (x_min_, y_min_) by the analytical framework of Polovnikov and Starkov [6] yielding loop density = 1/T. The fit selected the closest point on a grid of theoretical dip coordinates with T = 60–400 kb in 1 kb steps and $\:{v}_{0}^{eff}$ = 0.1–20 kb in 0.3 kb steps. Because the dip position is resolved at 1 kb, a one-bin shift changes the fitted T by roughly 7% for dips near 15 kb and 2.5% near 40 kb. At high depth, subsampling CVs of a few percent therefore reflect how often the dip moves by one bin. The dip was never found at the edge of its search window. The peak was at the window edge in 2 of 300 subsamples at 1 M read pairs and in none at 10 M or deeper. The fitted $\:{v}_{0}^{eff}$ reached the upper edge of the grid in 6 of 300 subsamples at 1 M (five for KBM7, one for hESC-FA-DpnII-R1-T2) and in none at 10 M or deeper. No subsample was excluded.

### Compartment analysis

Compartment eigenvectors were computed at 100 kb resolution using cooltools.eigs_cis [19] with GC-content phasing (hg38 GC track); the first eigenvector E1 was sign-oriented so that GC-rich bins are positive (A compartment). Saddle plots were computed with cooltools.saddle using 38 quantile groups, q_range = [0.025, 0.975]. Compartment strength was defined as (AA + BB) / (AB + BA) using the top/bottom 20% of E1-ranked groups. As a control, the saddle of every subsample was recomputed with bins ranked by the full-depth E1 of the same library. For each subsample we recorded the Pearson and Spearman correlation of E1 with the full-depth E1 over shared valid bins, the fraction of bins with matching A/B sign, and the scalar compartment strength.

### Insulation and boundary analysis

Insulation scores [3] were computed at 10 kb resolution using cooltools.insulation [19]. The 100 kb diamond window is used throughout the main text and Li’s thresholding [21] for boundary calling. Boundary precision, recall, and F1 were computed relative to the full-depth boundary set with ± 1,2,3,5 bin tolerance. Boundaries were matched per chromosome by nearest neighbour: a test boundary is a true positive if a full-depth boundary lies within ±х bins, and each reference boundary is counted at most once, so multiple calls falling inside one tolerance window cannot inflate the match count.

### Statistical analysis and threshold estimation

Depth thresholds were defined relative to replicate noise floors. Two replicate levels were used. Biological replicates were independently prepared libraries of the same cell type and protocol, whereas technical replicates were independent sequencing runs of the same library. Biological replicate groups comprised the three Oksuz et al. pairs of independent libraries (H1-hESC FA + DSG DpnII, HFFc6 Hi-C 3.0 and HFFc6 FA + DSG DpnII) and the IMR90, K562 and KBM7 series of Rao et al. For Rao et al., one run was taken per independent biological sample (GEO sample); where several runs came from one sample, the largest one was used. To exclude runs much shallower than the subsampled libraries, only biological replicates of at least 100 M read pairs were included, which excluded five of six HMEC runs (the HMEC group was therefore not used), one IMR90 run, four K562 runs and one KBM7 run. Technical replicate groups comprised 7–9 sequencing runs of each of six Oksuz et al. libraries. All accessions, contact counts and group assignments are listed in Table S6. Variability floors were computed separately for biological and technical replicates using full-depth data. For metrics based on pairwise comparisons between tracks, such as the correlation coefficient and boundary precision, all pairwise comparisons were performed between the replicate libraries belonging to the same dataset, and the resulting values were averaged to obtain a single value for that dataset. Boundary precision was evaluated at each positional tolerance (± 1, 2, 3 and 5 bins), and replicate floors were calculated separately for each reported tolerance. For metrics returning a single value per replicate, such as loop size and density, compartment strength, boundary count, and mean boundary strength, the coefficient of variation (CV) of these metrics were computed for each dataset as the standard deviation (SD) divided by the mean across the replicates belonging to that dataset. The per-group values were then averaged across groups without weighting, so that each cell line or protocol contributes once. The *n* − 1 weighted mean and the median are given for comparison (Table S7, Fig. S4). For boundary recall between replicates, which has no test/reference direction, the mean of recall and precision was used. Insulation floors were computed separately for each diamond window. The depth requirement of a metric was defined as the depth at which the subsample variability matches the biological-replicate floor. When single-value metrics are plotted as percentages of their full-depth values, the corresponding floor is shown as a band of 100% ± the averaged CV.

The CV of loop density and loop size was computed within each library. At every depth the mean and the sample SD were taken across the independent subsamples drawn at that depth from one library, and the coefficient of variation was their ratio, *CV = SD / mean*. The value reported against depth is the mean of these per-library CV across the libraries included in the analysis and the shaded band is the SD across the libraries. The full-depth point was set to zero, one full-depth measurement existing per library.

Pearson correlations for the E1 eigenvector and for the insulation track were computed between each subsample of each depth and the full-depth file of the same library. For that the two tracks were inner-joined on bin coordinates (chromosome, start, end), bins with a missing value in either track were discarded, and the coefficient was computed over the remaining bins. Insulation correlations were computed separately for the 50 kb and the 100 kb window. Because multiple independent downsampling replicates were generated at each depth, the mean correlation was taken within the library at each depth, then mean and SD across libraries were computed.

Threshold depths were estimated by log-linear interpolation, since sequencing depth was sampled at discrete values and is shown on a logarithmic scale. For each metric, the threshold was defined by the closest pair of consecutive depths whose mean values lay on opposite sides of the biological floor. If *x*_*k*_ and *x*_*k+1*_ denote these depths and y_k_ and y_k+1_ the corresponding metric values, the crossing depth *x* was obtained by interpolation $\:{log}_{10}\left(x\right)={log}_{10}\left({x}_{k}\right)+t\left({log}_{10}\right({x}_{k+1})-{log}_{10}({x}_{k}\left)\right)$, where $\:t\:=\:\frac{y{\:}_{k}\:-\:floor}{y{\:}_{k}\:-\:y{\:}_{k+1}\:}$. The same interpolation was applied to the upper and lower edges of the ± 1 SD band across libraries; the two depths obtained describe the spread between libraries, not a confidence interval of the crossing.

## Results

### Datasets and subsampling strategy

We analysed twelve Hi-C libraries drawn from two studies (Table 1). Eight libraries are from Oksuz et al. [7]: four from human embryonic stem cells (H1-hESC) and four from human foreskin fibroblasts (HFFc6), prepared with either a single formaldehyde cross-linker (FA) or the dual DSG + FA cross-linker protocol, with either DpnII alone or a DdeI+DpnII dual digest. In the naming of Oksuz et al., R denotes the biological replicate and T the technical replicate, so hESC-FA-DpnII-R1-T1 and R1-T2 are two sequencing runs of the same library; the twelve libraries therefore represent eleven independent preparations. The remaining four libraries are from four Rao et al. [1] cell lines (HMEC, IMR90, K562, KBM7), all prepared with in situ Hi-C.

The loop-density and loop-size analyses use all twelve libraries, because these metrics are derived from the genome-wide-averaged *P(s)* and their full-depth reference is internal to each library. For the compartment, insulation, and boundaries analyses, only nine libraries were retained: the eight Oksuz et al. libraries plus KBM7, because these metrics are normalised to a per-library full-depth value, and a meaningful cross-library aggregate requires comparable full depths (350-390 M). The three excluded Rao cell lines have full-depth read counts of 240 M (IMR90), 456 M (HMEC), and 591 M (K562), all outside the range of the Oksuz libraries.

For each library, the full FASTQ files were subsampled to six target depths: 200 M, 100 M, 50 M, 25 M, 10 M, and 1 M read pairs. At each depth we generated independent random subsamples to quantify stochastic variability: 25 at 1 M, 20 at 10 M and 25 M, 10 at 50 M, 5 at 100 M and 4 at 200 M. The full single library serves as the reference.

**Table 1:** Hi-C libraries used for the sequencing-depth benchmark. The dataset collection comprises twelve publicly available human Hi-C libraries spanning multiple cell lines, chromatin conformation capture protocols, restriction-enzyme strategies, and full-library sequencing depths

Dataset	SRA run accessions	Cell line	Protocol	Digestion	Depth, read pairs	Read length
hESC-DSG-DpnII-R1-T1^1^	SRR13601502	H1-hESC	FA + DSG; DpnII	DpnII	366 M	2 × 50
hESC-DSG-DpnII-R2-T1^1^	SRR13601511	H1-hESC	FA + DSG; DpnII	DpnII	357 M	2 × 50
hESC-FA-DpnII-R1-T1^1^	SRR13601520	H1-hESC	FA; DpnII	DpnII	357 M	2 × 50
hESC-FA-DpnII-R1-T2^1^	SRR13601529	H1-hESC	FA; DpnII	DpnII	353 M	2 × 50
HFFc6-DSG-DdeI-DpnII-R1-T1^1^	SRR13601574	HFFc6	Hi-C 3.0	DdeI+DpnII	361 M	2 × 50
HFFc6-DSG-DdeI-DpnII-R3-T1^1^	SRR13601583	HFFc6	Hi-C 3.0	DdeI+DpnII	368 M	2 × 50
HFFc6-DSG-DpnII-R1-T1^1^	SRR13601592	HFFc6	FA + DSG; DpnII	DpnII	366 M	2 × 50
HFFc6-DSG-DpnII-R2-T1^1^	SRR13601599	HFFc6	FA + DSG; DpnII	DpnII	352 M	2 × 50
HMEC	SRR1658680	HMEC	in situ Hi-C	MboI	456 M	2 × 101
IMR90	SRR1658676	IMR90	in situ Hi-C	MboI	240 M	2 × 101
K562	SRR1658694	K562	in situ Hi-C	MboI	591 M	2 × 101
KBM7^1^	SRR1658708	KBM7	in situ Hi-C	MboI	388 M	2 × 101

All libraries were included in the genome-wide *P(s)*-based analyses of loop-density and loop-size-associated features. Libraries marked with superscript 1 were additionally included in compartment, insulation-score and boundary analyses, for which comparable full-depth coverage was required for cross-library aggregation

### Stability of cohesin-mediated loop size and loop density across sequencing depths

The contact probability *P(s)* is the mean contact frequency between loci separated by genomic distance *s*, averaged over the genome. Across the genomic range typically probed by Hi-C, *P(s)* decays approximately as a power law, reflecting the fractal, crumpled organization of interphase chromatin [2, 22, 23]. At very large *s* close to 10-100 Mb, it flattens as loci become confined within chromosome territories [23], whereas at very short *s* about 1-10 kb, it is shaped by the finite physical resolution of the Hi-C protocol, including cross-linking efficiency and restriction-fragment size [6]. At intermediate scales, chromosome statistics are largely described by a crumpled polymer folded into cohesin-mediated loops by active loop extrusion [5, 24, 25]. Local deviations from scale-free behaviour, reflected as undulations in the log-derivative $\:dlogP\left(s\right)/dlogs$, therefore provide a sensitive readout of loop-associated chromatin architecture.

Two such deviations are central to this study: a local minimum (“dip”) at separations about 10–40 kb, and a local maximum (“peak”) at about 100 kb (Fig. 1). The dip arises from the interplay between the loop density and the finite Hi-C capture radius, so its position and depth jointly encode the loop density (Fig. S1) [6]. The peak, by contrast, reflects the characteristic loop size itself, largely independent of how densely loops are packed 5]). Because the two features respond to different structural parameters, tracking them separately allows us to ask whether subsampling degrades the inference of loop density and of loop size at the same rate, or not. The log-derivative of *P(s)* was computed for each subsample and both the loop-density-dip and the loop-size peak were extracted (Fig. 1). For each dataset, Tables S1, S2 report the full-depth loop parameters together with the subsample-to-subsample scatter of the loop density as depth decreases.

**Fig. 2:**
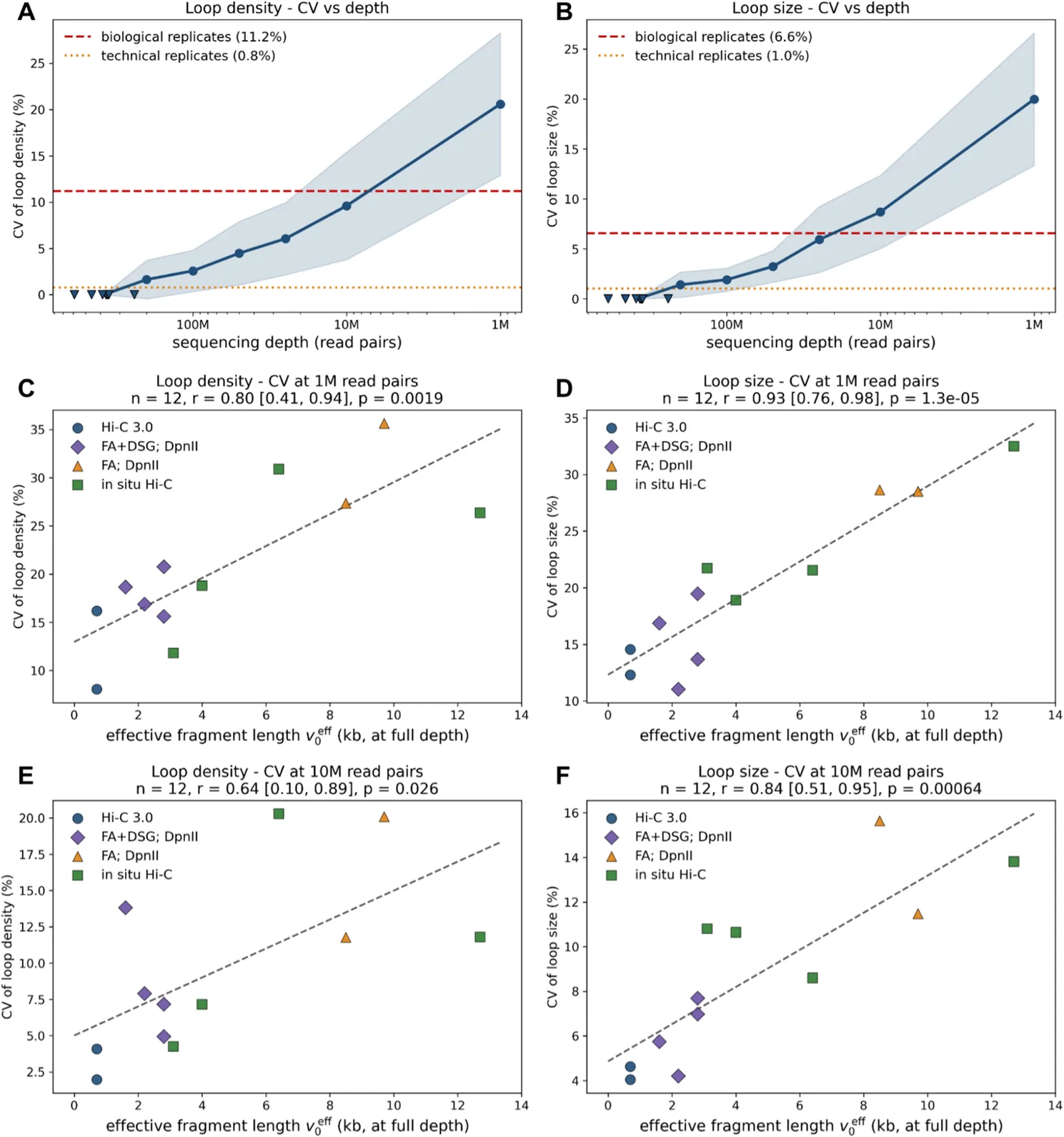
Stability of loop density and loop size under subsampling. Left column: loop density (inferred from the "dip"). Right column: loop size ("peak" position). (**A, B**) Coefficient of variation (CV) of loop density and loop size versus sequencing depth. For each library, CV was computed across independent subsamples drawn at a given depth, the curve shows the mean of these per-library CVs and the shaded band indicates SD across libraries. Dashed lines mark the biological-replicate (11.2% density, 6.6% loop size) and technical-replicate (0.8% density, 1.0% loop size) CV floors, computed as the unweighted mean of per-group CVs at full depth (Table S3). The full-depth point is plotted at the mean library depth. Inverted triangles along the x-axis mark the original sequencing depth of each library. (**C, D**) The same subsample CV at 1M read pairs, resolved per library, versus the effective fragment length $\:{v}_{0}^{eff}$ (inferred from the dip at full depth); each point is one library, coloured by protocol, dashed line – least-squares fit (Pearson r = 0.8, 95% CI [0.41, 0.94], p < 0.05) for density and r = 0.93, 95% CI [0.76, 0.98], p < 0.05 for loop size). (**E, F**) The same at 10M read pairs (Pearson r = 0.64, 95% CI [0.10, 0.89], p = 0.026 and r = 0.84, 95% CI [0.51, 0.95], p < 0.05, respectively). Libraries with smaller $\:{v}_{0}^{eff}$ give more stable low-depth estimates

Before examining the subsampling effects, we first establish two natural noise floors against which subsampling-induced scatter can be compared: the variability between independent biological replicates of the same condition, and the variability between independent technical replicates (Fig. 2A, B; Table S3). Biological replicates are independent Hi-C libraries prepared from separate cell populations of the same line, whereas technical replicates are independent re-sequencing runs of the same library. The former therefore capture both biological and experimental variation, the latter only sequencing noise. Measured at full library depth, the mean coefficient of variation (CV, the ratio of the standard deviation to the mean) across replicates is 11.2% (biological) and 0.8% (technical) for loop density, and 6.6% (biological) and 1.0% (technical) for loop size (Table S3). The biological CV varies between groups; IMR90 is the most variable (26% for density, 23% for loop size, against 1.4–12.5% in the other groups), and the thresholds obtained with alternative summaries of the floor are given in Table S7 and Fig. S4.

We find that for all twelve datasets the mean loop density and mean loop size remain within 6% of their full-depth values down to 10 million reads (Fig. 1; Table S1, S2). At 1 M, loop size stays within 9%, whereas loop density deviates by up to 15% in two libraries (HFFc6-DdeI-DpnII-R1-T1 and KBM7). Importantly, for both metrics, sequencing depth primarily affects the variation across the subsamples rather than the mean. To place these trends in context, we compare the subsampling-induced scatter to the replicate noise floors established above (Fig. 2A, B). For loop density, the subsampling CV grows from approximately 1.6% at 200 M to 4.5% at 50 M and 20.6% at 1 M reads. It reaches the biological-replicate floor (11.2%) at 7.2 M read pairs (range between libraries: 1.5–20.3 M), below which a single subsampled library is noisier than independent biological replicates at the full depth. For loop size, the subsampling CV crosses the biological-replicate floor (6.6%) at 20.2 M read pairs (range: 6.5–38.0 M). For neither metric does the subsampling CV fall to the technical-replicate floor at any tested depth. Even at 200 M reads the subsample-to-subsample CV (1.6% for density, 1.4% for loop size) remains above the CV between re-sequencing runs of the same library (0.8% and 1.0%). In practical terms, loop-density estimates from a single library remain within the biological floor down to approximately 7 M reads. At 10 M, the subsampling uncertainty (0.8 loops/Mb on average across libraries; Table S1) is already comparable to the spread between independent biological replicates of the same cell line (0.3–1.0 loops/Mb in five of six groups and 1.8 loops/Mb in IMR90; Table S3). Loop size is somewhat more demanding: its subsampling CV reaches the biological floor at 20 M reads, where the subsampling scatter (about 5 kb at 25 M; Table S2) matches the spread between biological replicates (1.4–5.9 kb in five of six groups, 20 kb in IMR90), and only at 1 M does the scatter (10–32 kb) become large relative to the approximately 35 kb spread of loop sizes observed between cell types. Below these depths the subsampling variation exceeds the natural biological variability for both metrics, and a single subsample is a noisier estimate of the underlying parameter than an independent biological replicate sequenced to full depth.

Interestingly, the subsampling uncertainty of both loop-density and loop-size estimates is strongly affected by the experimental protocol, which can be summarized in the effective capture radius at which two loci are registered as a contact in the experiment [6]. We express this radius in genomic units as the effective fragment length $\:{v}_{0}^{eff}$, which can be inferred directly from the full-depth dip position (Fig. 2C-F). Across libraries, $\:{v}_{0}^{eff}$, is associated with low-depth robustness (Fig. 2C–F): protocols with tighter capture radius (such as Hi-C 3.0), corresponding to smaller $\:{v}_{0}^{eff}$, yield more stable estimates under subsampling. Consistently, the HFFc6 DdeI+DpnII libraries, with $\:{v}_{0}^{eff}\approx\:0.7$ kb, show the smallest scatter at 10 M read pairs (0.14–0.32 loops/Mb). In contrast, the FA-crosslinked hESC libraries and KBM7 with $\:{v}_{0}^{eff}\approx\:8.5-13$kb show the largest uncertainty, reaching up to approximately 3.1 loops/Mb at 1 M read pairs.

### Compartment eigenvectors and strength

**Fig. 3:**
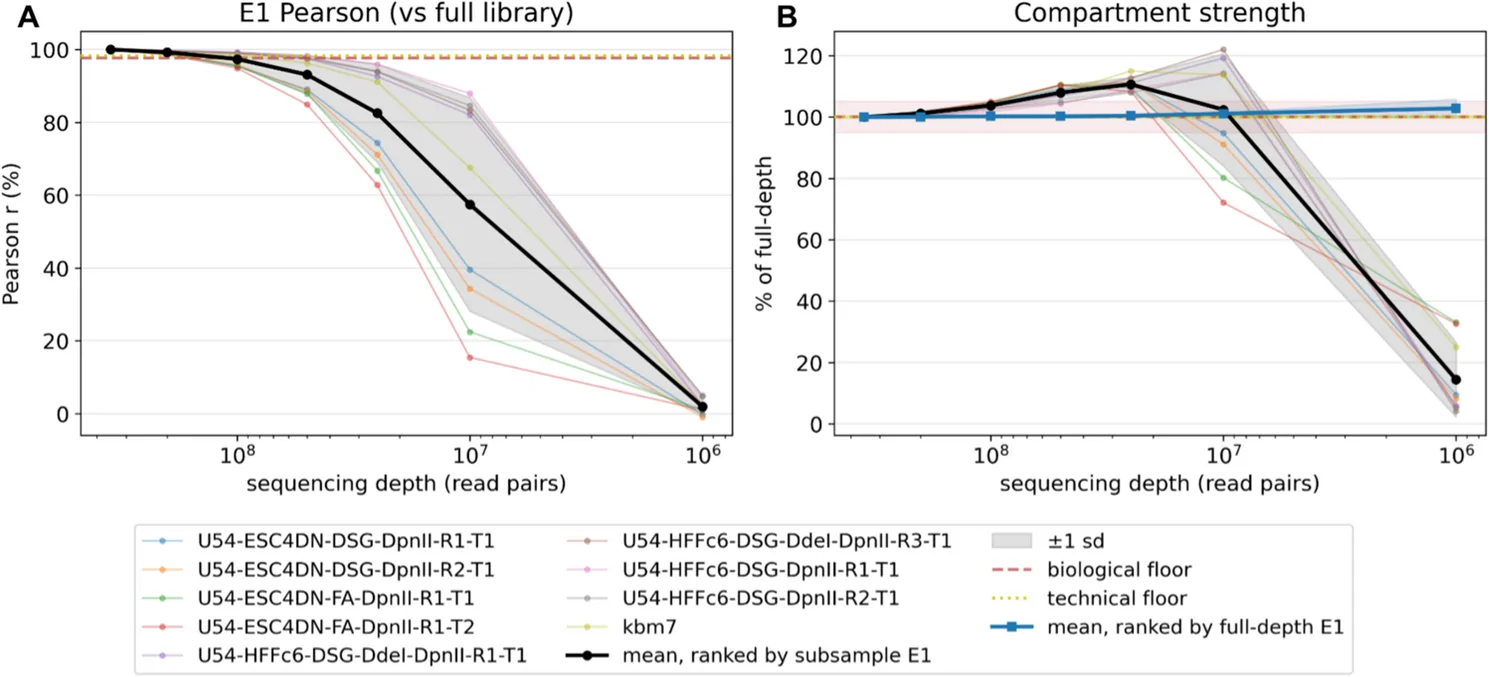
Compartment metrics as a function of sequencing depth (9 libraries). Each panel shows a metric vs subsampling depth. The bold black line is the cross-library mean, the gray band indicates ± 1 s.d. Red and yellow horizontal lines mark the biological- and technical-replicate floors, respectively. (**A**) E1 Pearson correlation with the full-depth eigenvector. (**B**) Compartment strength as % of full. Black: bins ranked by the E1 of each subsample, showing inflation at intermediate depths and collapse at 1M; blue: bins ranked by the full-depth E1 of the same library, which remains near 100% at all depths

Next we turn to the question of compartment stability with respect to downsampling. A/B compartments were computed at 100 kb resolution across the nine libraries with comparable full depth. The metrics are summarised in Fig. 3 (per-depth values in Table S4). We find that the compartment eigenvector is relatively preserved down to approximately 50 M read pairs (E1 Pearson *r* = 93% of full library, Fig. 3A), begins to degrade substantially at 25 M (83% correlation), and approaches random expectation at 1 M read pairs (correlation near zero, sign agreement about 51%).

To place these depth-dependent changes in the context of natural sample variability, we computed pairwise E1 correlations between independent biological replicates (six groups, Table S6) and between technical replicates (7–9 re-sequenced runs of each of six Oksuz et al. libraries). The mean E1 Pearson r equals 97.7% between biological replicates and 98.3% between technical replicates: independent deep libraries reproduce the E1 track nearly as closely as re-sequencing runs of one library. The subsampled E1 Pearson crosses the biological-replicate floor at 111.5 M (range between libraries: 40.2–159.5 M) read pairs: below this depth the depth-induced noise exceeds the biological variability (Fig. 3A). The technical-replicate floor is crossed at approximately 140 M. These estimates reveal a much higher sequencing depth required for the robust compartmental annotation than in the case of loop characteristics analysed above.

**Fig. 4:**
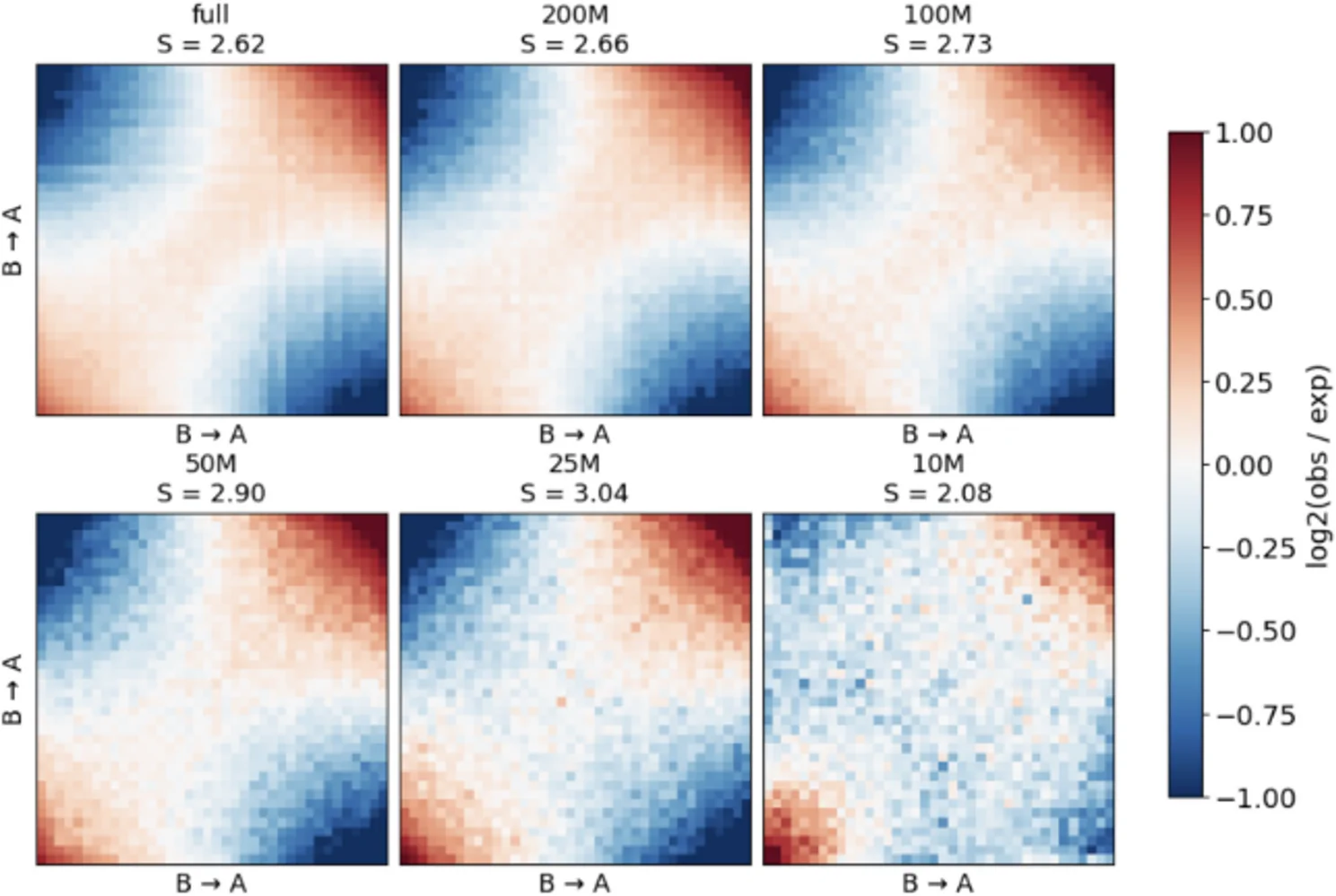
Saddle plots of observed/expected contact enrichment ranked by E1 eigenvector, for one ESC dataset (DSG-DpnII) at six sequencing depths (full library, 200M, 100M, 50M, 25M, 10M; one subsample per depth). Bins are ranked by the E1 of the same subsample. Red – enriched cis-compartment contacts; blue – depleted trans-compartment contacts

Compartment strength exhibits an unusual behaviour (Figs. 3B and 4): when bins are ranked by E1 of the same subsample, it rises with downsampling (from 100% to approximately 111% of full at 25 M) before dropping to approximately 14% at 1 M. When the same bins are ranked by the full-depth E1, compartment strength stays within 100–103% of its full-depth value at every depth, down to 1 M (Fig. 3B, blue). Both the inflation and the collapse therefore arise from ranking bins with a noisy eigenvector computed from the same data: randomly emerging extreme bins are placed in the saddle corners, and at 1 M the ranking becomes essentially random. The compartment strength computed with the original bin ranking, by contrast, is preserved down to 1 M. The biological CV of compartment strength across replicate groups was 5.1% (red band in Fig. 3B), the technical CV 0.4% (yellow band).

The aggregate value at 10 M, however, hides a divergence between libraries: the four hESC libraries decline to about 84% of full, whereas the five non-hESC libraries (HFFc6 and KBM7) rise to about 117% (Figs. 3B, 102.4% across all nine, Table S4). The separation follows cell type rather than protocol: the H1-hESC libraries cluster together regardless of FA or FA + DSG crosslinking, as do the HFFc6 libraries. It does not, however, reflect a difference in compartmentalization. With bins ranked by the full-depth E1, all nine libraries stay within 100–102% of their full-depth strength at 10 M. The divergence instead tracks how strongly the subsample eigenvector degrades: at 10 M, the hESC libraries retain an E1 correlation of only 15–40% with full depth, against 68–88% for the other libraries, and within each group, libraries with a lower E1 correlation show a lower apparent strength. Where E1 is largely lost, random re-ranking of bins lowers the apparent strength; where it is preserved, the selection of chance extremes raises it.

### Insulation score and boundary calling

**Fig. 5:**
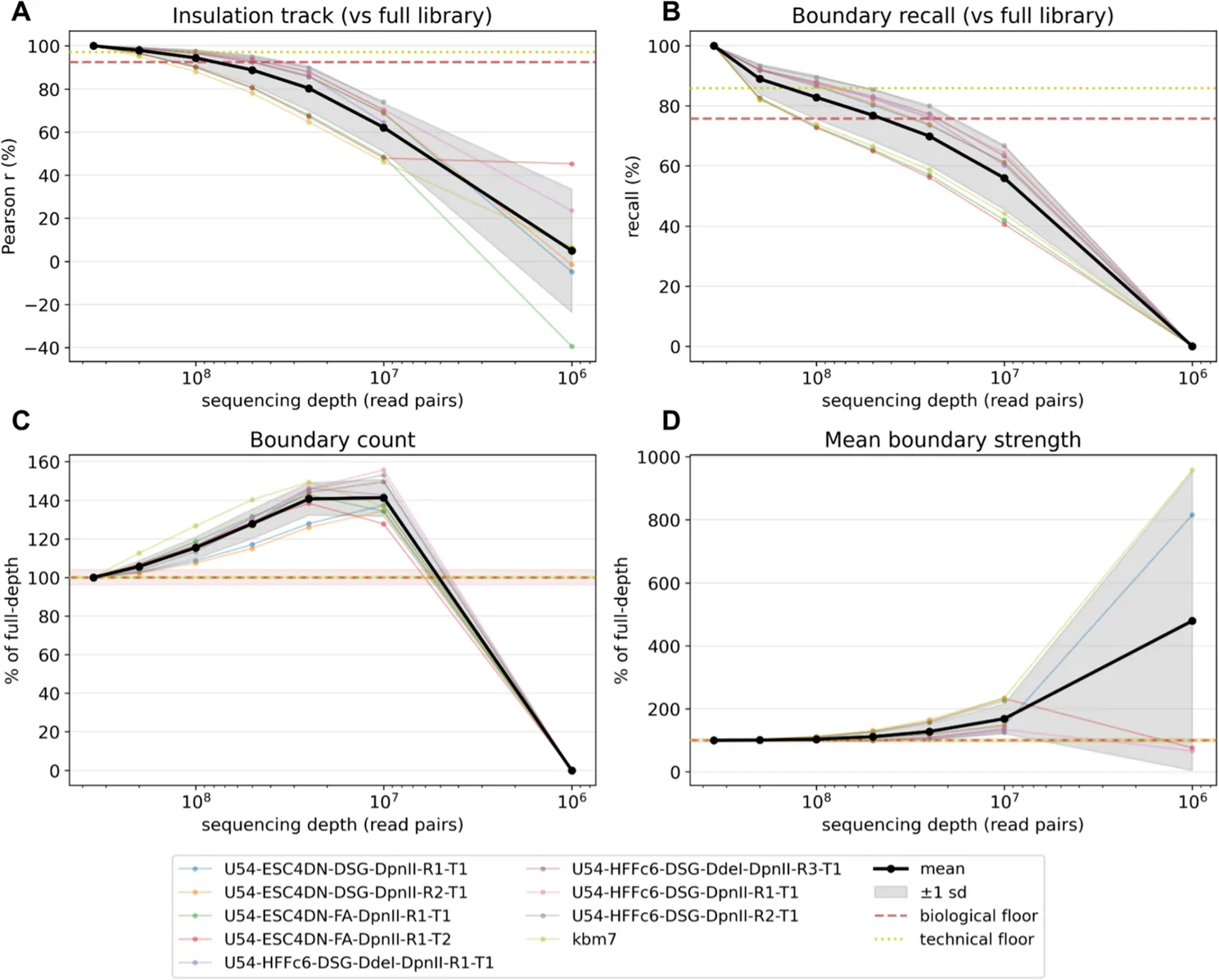
Insulation and boundary metrics as a function of sequencing depth (n = 9 libraries, 100 kb window, 10 kb resolution). Thin lines: individual libraries; bold: mean ± 1 s.d. Red and yellow lines: biological- and technical-replicate floors. (**A**) Pearson correlation of the insulation track with the full-depth track. (**B**) Boundary recall vs the full-depth boundary set (±2-bin tolerance). (**C**) Number of called boundaries and (**D**) mean strength of called boundaries as % of full, both showing inflation at intermediate depths

To probe the stability of Hi-C domains across sequencing depths, genome-wide insulation scores and domain boundaries were computed at 10 kb resolution and 100 kb window. These boundaries were not filtered to any specific size so they demarcated both topologically-associated domains and compartmental/subcompartmental domains. For each subsample, we evaluated Pearson correlation of the insulation track with the full-depth reference, as well as the relative number of called boundaries, relative mean boundary strength, and relative boundary recall (Fig. 5, per-depth values in Table S5).

Similarly to E1 correlation (Fig. 3A), insulation-score correlation degrades substantially already at high sequencing depths: 98% remained at 200 M, 89% at 50 M, and 62% at 10 M. At 1 M reads the correlation is virtually zero. The biological-replicate floor for insulation-score correlation is lower than for compartments: the mean Pearson r between biological replicates at full depth was 0.92 (technical 0.97), against 0.98 for E1. This is consistent with the higher intrinsic stochasticity of submegabase domains, which dominate the insulation track, compared with the more persistent compartmental boundaries (Fig. 3A). The subsampling curve crosses the biological floor at 78.3 M (range 31.7–124.1 M) read pairs, about 1.4-fold less than the 111.5 M critical depth for compartments. The 50 kb window gives a nearly identical threshold (78.4 M, range 36.1–127.5 M; floor *r* = 0.83; Fig. S2).

Furthermore, the number of called boundaries (Fig. 5C) and mean boundary strength (Fig. 5D) show a non-monotonic inflation pattern similar to compartment strength: boundary count rises to 141% of full at 25 M as at intermediate depths noise creates spurious insulation dips that pass the threshold and inflate the count. Mean boundary strength, averaged over called boundaries, inflates in parallel, rising from approximately 101% of full depth at 200 M to 128% at 25 M and 169% at 10 M (Fig. 5D). At 1 M, boundaries were still called in only 4 of 9 libraries, in numbers below 0.1% of the full-depth count, and their strength is not interpretable (Table S5). The inflation is a selection effect of the same kind as for compartment strength: boundaries are called and scored on the same noisy track, so the minima that pass the threshold are preferentially those deepened by chance. The effect is amplified at smaller diamond windows, where fewer pixels enter each insulation value: with a 50 kb window, insulation-track correlation and boundary recall follow trends similar to those observed with the 100 kb window (Fig. S2A, B), whereas mean boundary strength increases substantially, reaching approximately 250% of the full-depth value at 10 M reads (Fig. S2D), likely for the same reasons underlying the inflation of compartment strength (Fig. 3B).

At 1 M reads the insulation track becomes essentially flat (its correlation with the full-depth track is undefined in 3 of 9 libraries). The net effect is captured by boundary recall, which at ± 2-bin tolerance drops from 89% at 200 M to approximately 56% at 10 M (Fig. 5B) and reaches the biological floor (75.8%) at 44.9 M read pairs (range 19.2–101.6 M). With floors computed separately for each tolerance, the crossing moves to shallower depth as the tolerance widens: 58.7 M at ± 1, 34.3 M at ± 3 and 21.2 M at ± 5 bins (Fig. S3).

### Sequencing cost implications

To translate depth thresholds into real budgets, we note that a library at depth d (in million read pairs) sequenced at 2 × 150 bp, the read length most commonly ordered today, generates approximately 0.3·d Gb of raw sequence (2 × 150 bp × d × 10⁶). The libraries analysed here were sequenced at 2 × 50 bp (Oksuz et al.) and 2 × 101 bp (Rao et al.); longer reads map more reliably, so the thresholds in raw read pairs are likely conservative for 2 × 150 bp sequencing. Hi-C libraries are compatible with any Illumina paired-end instrument. We price the tiers below assuming a representative $3 per Gb for 2 × 150 bp reads on the NovaSeq X. (Table 2) [26, 27].

We recommend at least 10 M read pairs for loop-density and 20 M for loop-size inference from a single library, roughly 3–6% of the depth of a full library and of its sequencing cost. For protocols with a broad capture radius (FA-only crosslinking or in situ Hi-C with large $\:{v}_{0}^{eff}$), about 50 M read pairs are advisable for loop size, since the per-library thresholds for these libraries reach 43–47 M. At these depths, both the density CV and the loop-size CV remain within or close to the biological-replicate floor, so further depth yields diminishing returns relative to the natural variability between independent cell populations. These recommendations assume high-complexity libraries such as those analysed here; low-input or low-complexity libraries may carry additional variance from duplication and limited unique molecules that read subsampling does not model, and may require deeper sequencing (see limitations).

**Table 2:** Estimated Illumina sequencing costs per metric tier (2×150 bp paired-end reads)

Target metric	Reads (M)	Raw Gb	Est. cost	
Loop density	10	3	≈$9	
Loop size	20	6	≈$18	
Insulation	80	24	≈$72	
Compartments	110	33	≈$99	
Standard Hi-C	350	105	≈$315	

Costs are sequencing reagents only at $3 per Gb (2×150 bp); library preparation, indexing, quality control and overheads are not included. Depth estimates correspond to the biological-replicate floor of each metric, rounded up (measured thresholds: 7M, 20M, 78M and 111M), i.e. the depth below which subsampling noise exceeds the variability between independent biological replicates. Sequencing beyond this depth improves precision that is not distinguishable from biological variability

## Discussion

Since the introduction of Hi-C, sequencing depth has increased by orders of magnitude. The first Hi-C maps, generated from fewer than 10 million informative read pairs, were already sufficient to reveal the large-scale A/B compartmentalization of the genome [2], whereas later high-depth in situ Hi-C maps resolved domains, loops and finer-scale compartmental structure [1]. Modern bulk Hi-C libraries are now routinely sequenced to hundreds of millions of read pairs, producing tens to hundreds of gigabases of raw sequence [7, 8, 28]. This increase in depth has transformed Hi-C from a genome-wide assay that primarily revealed broad folding patterns into a quantitative, high-dimensional readout from which chromatin organization can be analysed across multiple genomic scales.

At the same time, the interpretation of Hi-C maps has become increasingly compressed. Many questions about chromosome folding can now be formulated in terms of a limited number of biophysical readouts, such as parameters of cohesin-mediated random loops (mean loop size and linear density), compartment eigenvectors, compartment strength, insulation profiles and boundary positions. These metrics reflect different scales of genome organization and rely on different amounts of averaging across the contact map. Compartment annotations capture megabase-scale segregation of active and inactive chromatin [1, 2]. Insulation profiles quantify local separation between neighbouring genomic domains [3]. Loop-extrusion models connect local deviations in the contact probability *P(s)* to loop-scale physical parameters [4–6, 24, 29, 30]. In this sense, computational and biophysical analysis of Hi-C performs a form of dimensionality reduction: a large contact matrix is projected onto a small number of meaningful structural readouts.

This motivates the central practical question addressed here: how much sequencing depth is required for each Hi-C-derived metric? This question remains relevant despite the continuing increase in sequencing throughput and reduction in per-base cost [27]. Hi-C library preparation introduces a substantial fixed per-sample cost, whereas sequencing depth is the main variable cost that linearly scales with the desired number of read pairs. Therefore, reducing depth does not reduce the total experimental cost proportionally, but it can substantially lower the sequencing component and make larger designs with more conditions, perturbations or biological replicates more feasible (Table 2). Importantly, there is no single universal depth requirement. Sequencing-depth requirements depend not only on the genomic scale of the feature, but also on the extent of genomic averaging, the Hi-C protocol and, most importantly, how reproducible the same metric is between independent biological replicates. Our benchmark therefore avoids defining “sufficient depth” as an arbitrary fraction of the full-depth value. Instead, we use an empirical biological floor: the variability observed between independent biological replicates of the same cell line at full depth. This floor approximates the accuracy limit of the metric under the present experimental conditions, because no amount of additional sequencing can eliminate genuine variation between independently prepared cell populations.

**Fig. 6:**
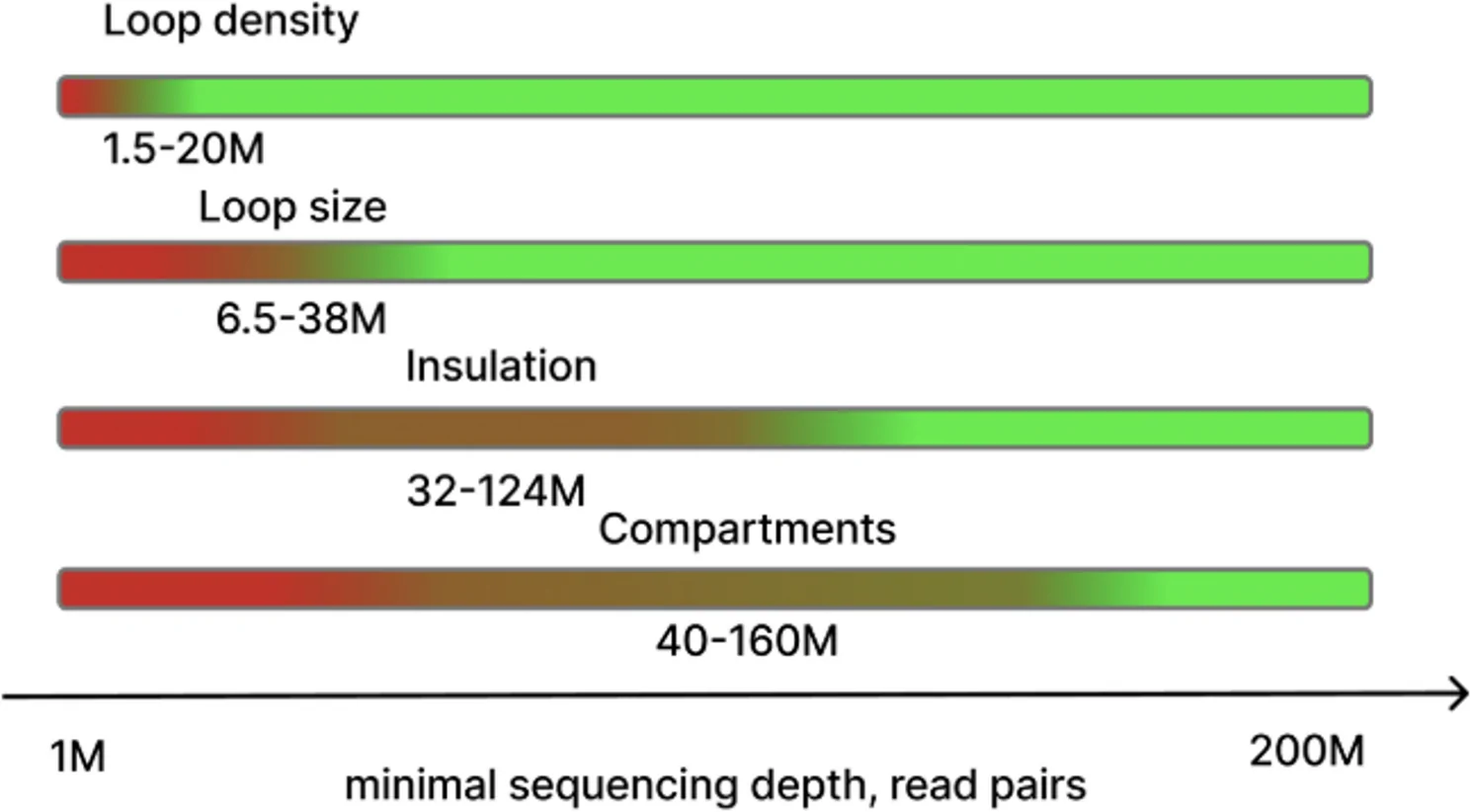
Hierarchy of critical sequencing depths across Hi-C metric families. Schematic, not to scale. Colours indicate whether a depth is sufficient: red, below the range for all libraries; brown, within the range between libraries, where sufficiency depends on the library and protocol; green, above the range for all libraries. For loop density and loop size, critical depth was defined as the point at which the mean subsampling CV crossed the corresponding full-depth biological-replicate CV floor. For compartment eigenvectors and insulation profiles, critical depth was defined as the point at which the mean correlation with the full-depth track crossed the corresponding full-depth biological-replicate correlation floor. Central values were obtained by log-linear interpolation of the mean curve between the two analysed depths bracketing the floor; ranges show the spread between libraries (interpolation of the ±1 SD envelope), not confidence intervals

On this criterion, the depth hierarchy emerges (Fig. 6). The *P(s)*-derived loop-density dip is the most robust metric, reaching the biological floor at approximately 1.5–20 M read pairs. The loop-size-associated peak reaches its biological floor at 6.5–38 M. Insulation profiles reach the corresponding floor at roughly 32–124 M read pairs. Compartment eigenvectors, despite representing large-scale genome organization, are the most demanding under this criterion, crossing their biological-replicate floor at 40–160 M read pairs. Thus, the ordering reflects the combination of two effects: how strongly a metric degrades under subsampling, and how reproducible that metric is between independent full-depth biological replicates.

A key point is that the mean of many downsampled libraries is not by itself a useful definition of sufficient depth. For the *P(s)*-derived loop metrics, the mean estimate remains close to the full-depth value even after severe downsampling to 1 M reads (see Fig. 1; Tables S1, S2). This is expected: averaging over many independent low-depth subsamples effectively reconstructs a deeper experiment. In practice, however, a researcher who deliberately sequences at low depth obtains one random sample of reads, not an ensemble average over many such samples. The relevant question is therefore how far a single low-depth library can deviate from the full-depth estimate. This is why subsample-to-subsample scatter, rather than the mean alone, provides the appropriate measure of reliability.

The loop-density and loop-size stability analysis in Fig. 2 illustrates this distinction. Across all twelve libraries, the mean loop density and mean loop size remain within approximately 6% of their full-depth values down to 10 M reads. Sequencing depth primarily affects the scatter around these means. Loop density is a global steady-state parameter: in the extrusion picture, it is determined by the total number of chromatin-bound cohesins per genomic length. Its subsampling coefficient of variation increases from approximately 1.6% at 200 M reads to 4.5% at 50 M and 20% at 1 M reads and reaches the biological-replicate floor of 11.2% at approximately 7 M reads. Loop size is different: it is an emergent length scale shaped by cohesin processivity together with the distribution and kinetic properties of barriers to loop extrusion [31]. Its biological floor is lower, 6.6%, indicating that the inferred loop-size peak is more stable across independent biological replicates than the inferred loop density. This stronger biological reproducibility leaves less room for sequencing-induced scatter, and the biological floor is therefore reached only at approximately 20 M reads; the technical floor is not reached at any tested depth. Thus, loop density can be estimated from relatively shallow libraries, whereas loop size requires somewhat deeper sequencing to remain within its narrower natural biological variability.

The robustness of loop-scale inference also depends strongly on experimental protocol. The relevant parameter is the effective fragment length $\:{v}_{0}^{eff}$, which expresses the Hi-C capture radius in genomic units and can be inferred from the full-depth dip position [6]. Libraries with smaller $\:{v}_{0}^{eff}$ have sharper dip features and tolerate downsampling better (Fig. 2C, E). In our data, the HFFc6 DdeI+DpnII libraries, with $\:{v}_{0}^{eff}$ close to 0.7 kb, show the smallest loop-density scatter at 10 M reads (0.14–0.32 loops/Mb). In contrast, broader-capture libraries, including FA-crosslinked hESC libraries and in situ Hi-C libraries such as KBM7 and IMR90, have larger effective fragment lengths, up to approximately 13 kb, and show substantially greater uncertainty, reaching approximately 3.1 loops/Mb at 1 M reads. Similar conclusions were reached in [7], where the number of detected loops in the data was found to be more sensitive to the amount of sequenced reads for protocols with a single cross-linking (FA) than in protocols with double cross-linking (FA + DSG). Protocol choice and sequencing budget are therefore coupled: tighter capture makes low-depth *P(s)*-based inference more stable, whereas broader capture requires deeper sequencing to reach the same precision. Note that in this study the critical read depths are provided as the averages across all available protocols (see Table 1).

The result that compartment eigenvectors require the deepest sequencing under our criterion may appear surprising. A/B compartments were discovered in the first Hi-C study using far fewer reads than modern maps [2]. The apparent contradiction comes from the difference between detecting the existence of compartments and quantitatively reproducing a high-resolution compartment track. The broad plaid-like compartment pattern, and even the sign of the E1 eigenvector, can be visibly non-random at low depth. This is sufficient to discover compartmentalization or classify the genome into broad A/B domains. Our benchmark asks a stricter question: at what depth does a 100 kb E1 eigenvector from one subsample reproduce its full-depth counterpart as well as independent full-depth biological replicates reproduce each other? Because the biological-replicate E1 correlation is very high, approximately *r* = 0.98 and close to the technical-replicate value, depth-induced noise must be very small before it becomes comparable to the biological floor. The saddle control supports this distinction: with bins ranked by the full-depth eigenvector, the compartment contrast in the contacts is preserved down to 1 M read pairs, whereas the eigenvector itself is lost. Thus, compartments can be qualitatively detected at low depth, but stable quantitative compartment annotation requires deeper sequencing.

This comparison also clarifies why insulation and boundary metrics reach their biological floor at lower depth than compartments, despite being more local and more visibly sensitive to coverage. Insulation-track correlation decays substantially under downsampling, falling to approximately 89% at 50 M reads and 62% at 10 M reads (Fig. 5A). However, the full-depth biological-replicate floor for insulation is lower than for compartments, approximately *r* = 0.92 against 0.98. In other words, local domain structure is less reproducible between independent biological libraries than the compartment eigenvector. Because the biological floor is lower, the subsampling curve reaches it at shallower depth, at around 80 M reads. The minimal useful depth of a metric is therefore not simply the level at which it captures a substantial fraction of the full-depth effect, but the depth at which sequencing noise becomes small relative to the biological variability of that metric.

Scalar summary statistics require special caution. Compartment strength, boundary count and mean boundary strength can appear to improve under downsampling even while the underlying contact map degrades (Fig. 3–5; Fig. S2). These increases should not be interpreted as stronger segregation. Rather, they arise when the features being scored are selected on the same noisy data. For compartment strength, bins are ranked by an eigenvector computed from the subsample itself, so bins whose contacts are extreme by chance populate the saddle corners; ranking by the full-depth eigenvector removes the effect entirely (Fig. 3B). For boundaries, the insulation profile becomes noisy at low depth and shows extra dips. The dips that happen to be deeper are more likely to be called as boundaries, so both the number of boundaries and their mean strength increase. Such summary values should therefore be compared between samples only at the same sequencing depth, or with compartments and boundaries defined on one common reference map. Other summary values that compare enriched and depleted signal, such as the average signal at loop anchors, may behave in the same way if the loops are called from the same data.

A simple genome-scale consideration helps interpret why depths near 10–25 M read pairs appear as natural lower limits for the most robust metrics. A haploid human genome contains approximately 3 billion base pairs. Ten million read pairs at 2 × 150 bp correspond to about 3 billion sequenced bases, i.e. roughly one haploid genome-equivalent of raw sequence. For the libraries analysed here, sequenced at 2 × 50 and 2 × 101 bp, one genome-equivalent is reached only near 30 M and 15 M read pairs, respectively. Genome copy number also differs between lines: KBM7 is near-haploid and K562 is aneuploid, so the coverage per genome copy differs at the same number of read pairs. This is not a true coverage threshold for Hi-C contacts: Hi-C reads are not uniformly distributed along the genome, contact maps sample pairs of loci rather than individual bases, and many bins are filtered due to mappability or balancing failures. Nevertheless, it provides useful intuition.

Below roughly one genome-equivalent of raw sequence, sparse sampling of genomic regions becomes unavoidable. This raises a related and more fundamental point: the biological-replicate floor may itself reflect a finite sampling of the chromatin conformational ensemble. Two independent populations of the same cell line are not identical realizations of one 3D genome; rather, each Hi-C library averages over a finite number of cells, loci and transient folding states. Similarly to sampling-induced variation during the downsampling procedure, biological replicate variability may therefore be interpreted, at least in part, as sampling of the ensemble of chromosome conformations available to that cell type. Our design does not separate this cell-to-cell heterogeneity from differences in library preparation: the biological floor combines both, whereas the technical floor, obtained by re-sequencing one library, bounds the part due to sequencing alone. The depth thresholds therefore do not require this separation: they mark where the noise added by limited sequencing becomes comparable to the total variability between independent samples.

The cost estimates make these exercises practical. With 2 × 150 bp paired-end sequencing, a library at depth d million read pairs generates approximately 0.3d Gb of raw sequence. At a representative NovaSeq X reagent cost of approximately $3/Gb [26, 27], the recommended depths correspond to sequencing reagent costs of approximately $9 for loop density at 10 M read pairs, $18 for loop size at 20 M read pairs, $72 for insulation at 80 M read pairs and $99 for compartment eigenvectors at 110 M read pairs. A standard 350 M-read-pair Hi-C library sequenced at 2 × 150 bp would require about 105 Gb of raw sequence and about $315 in sequencing reagents alone. These values exclude library preparation, indexing, quality control, labour and facility overheads, and should therefore be interpreted as lower-bound sequencing costs rather than full experimental costs. Nevertheless, the relative conclusion is robust: several Hi-C-derived metrics reach their biological-floor depth far below the depth of modern full-scale Hi-C libraries, so metric-specific depth selection can substantially reduce the sequencing component of the cost and make larger replicate- or perturbation-based designs more feasible.

Several limitations bound the scope of these recommendations. First, our benchmark is restricted to bulk Hi-C from human cell lines mapped to the human genome. The numerical thresholds should not be assumed to transfer directly to other genomes, especially genomes with different size, repeat content, mappability, chromosome number or restriction-fragment distribution. Second, the biological floors were estimated from deep libraries (at least 100 M read pairs) and represent a lower bound on biological-replicate variability under low sequencing noise. They are estimated from groups of two to seven replicates and vary between groups, IMR90 being the most variable, and the loop thresholds depend on how the groups are summarised; HMEC could not be included for lack of deep independent replicates; at reduced depth, variability between biological replicates would increase because each replicate would also accumulate independent sampling noise. Third, random subsampling models the loss of the read depth but not the loss of library complexity: subsamples are drawn from the same pool of unique molecules and inherit its duplication level, ligation efficiency and capture biases, so genuinely shallow experiments may vary more than our subsamples do. The same holds within the benchmark itself: subsamples of a given depth share reads with each other and with the full-depth reference against which they are compared; as a result, the scatter we observe is somewhat smaller than that of independent libraries at the same depth, and the final thresholds are correspondingly slightly shallow. This cannot be resolved by comparing subsamples with one another, which still measures read sampling within a single preparation. The only fully independent comparison is between separate libraries, and that is what the biological floors are built from. The effect is largest for compartments and insulation, whose thresholds are about 30% and 20% of a library, and negligible for the loop metrics, at 2–6%. Fourth, the benchmark covers standard bulk Hi-C only; we did not directly test Micro-C [32, 33], capture Hi-C [34] single-cell Hi-C [35–37] or other 3 C-derived assays. Fifth, the analysed libraries were sequenced at 2 × 50 and 2 × 101 bp; since longer reads map more reliably, the thresholds in raw read pairs are likely conservative for 2 × 150 bp sequencing, but they do not transfer exactly between read lengths. Sixth, our critical-depth thresholds are averaged across protocols, although both our results and previous work [6, 7] indicate that cross-linking and restriction strategies affect the effective capture radius and therefore the robustness of *P(s)*-derived metrics to downsampling. Seventh, part of the robustness of the *P(s)*-derived estimates may come from the estimator itself: the slope is smoothed and the dip is matched to the analytical model, which constrains its shape and damps noise. The loop thresholds therefore apply to this procedure and should not be assumed for other ways of extracting loop parameters. Finally, we did not test other metrics, such as the average signal at loop anchors, and insulation and boundary thresholds depend on caller settings, including bin size, diamond-window size, thresholding method and positional tolerance. For these reasons, the qualitative hierarchy in Fig. 6 is likely more robust than the exact numerical thresholds.

## Conclusion

Sequencing-depth requirements for genome-averaged Hi-C metrics are metric-specific and depend on both genomic averaging and biological reproducibility. Using replicate-to-replicate variability at full depth as an empirical biological floor, we find a clear hierarchy: genome-wide *P(s)*-derived loop density is the most robust metric, loop-size inference requires somewhat deeper sequencing, insulation and boundary metrics require intermediate depth, and 100 kb compartment eigenvectors require the deepest sequencing because of their higher biological-replicate reproducibility. These results argue against a single universal Hi-C depth standard and provide a practical framework for choosing sequencing depth according to the downstream metric, experimental protocol and intrinsic biological variability. They do not extend to the detection of individual loops or boundaries at specific loci, which requires substantially deeper sequencing [7, 14].

## Supplementary information

Below is the link to the electronic supplementary material.

Supplementary Material 1

## Data Availability

The public Hi-C datasets analysed in this study are available from the NCBI Sequence Read Archive under the accession identifiers listed in Table 1. The source code and analysis scripts are available at https://doi.org/10.5281/zenodo.23022391 and mirrored at https://github.com/agranovskiii/sequencing-depth-requirements. Additional processed summary tables used to generate the figures are included in the Supplementary Information.

## Abbreviations

A/B compartments: Active/Inactive chromatin compartments
P(s): Contact probability as a function of genomic separation s
CTCF: CCCTC—binding factor
FA: Formaldehyde
DSG: Disuccinimidyl glutarate
GC: Guanine—cytosine
E1: First eigenvector
CV: Coefficient of variation
PCR: Polymerase chain reaction
