# Supplementary material for: Systematic assessment of sequencing depth requirements for Hi-C-derived metrics
Source: BMC Genomics. 2026 Oct 15;27:860. doi: 10.1186/s12864-026-13455-7 (PMC13670550; doi:10.1186/s12864-026-13455-7)
Supplement: Supplementary file 1 [file 12864_2026_13455_MOESM1_ESM.docx]

# Supplementary

**Figure S1.** Fountain diagram of fitted *P(s)* derivative dip coordinates. Gray curves: analytically predicted positions for the inverse cohesin density T = 60–400 kb, shown every 5kb (step 1kb). Filled markers: experimentally fitted dip positions extracted from *P(s)* derivative. Colour encodes sequencing depth; marker shape identifies the dataset.

**Figure S2. Insulation metrics as a function of sequencing depth.** (n = 9 libraries, 50 kb window, 10 kb resolution). Thin lines: individual libraries; bold: mean ± 1 s.d. Red and yellow lines: biological- and technical-replicate floors. (A) Pearson correlation of the insulation track with the full-depth track. (B) Boundary recall vs the full-depth boundary set (±2-bin tolerance). (C) Number of called boundaries and (D) mean boundary strength as % of full, both showing non-monotonic inflation at intermediate depths. Floors are computed at the 50 kb window: r = 0.833 (biological) and 0.926 (technical) for the insulation track.

**Figure S3. Boundary recall across tolerance levels.** Each panel shows boundary recall (fraction of full-depth boundaries recovered) as a function of sequencing depth for a given positional tolerance: (A) ±1 bin (10 kb), (B) ±2 bins (20 kb), (C) ±3 bins (30 kb), (D) ±5 bins (50 kb). Thin lines represent individual libraries; the bold black line is the cross-library mean ± 1 s.d. Dashed red and dotted yellow lines mark the biological and technical floors, computed separately for each tolerance (biological: 67.9%, 75.8%, 79.7% and 84.0%; technical: 80.2%, 85.9%, 88.1% and 90.4% at ±1, 2, 3 and 5 bins). Increasing the tolerance progressively raises recall at every depth.

**Figure S4.** Sensitivity of the loop thresholds to the definition of the biological floor. As Fig. 2A,B, but the biological floor is the mean of per-group CVs weighted by n−1 (dashed red line; 16.9% for loop density, 12.8% for loop size), and the shaded band is ±1 weighted SD across groups. With this floor, the thresholds are 2.2M for loop density and 4.4M for loop size, compared with 7.2M and 20.2M for the unweighted mean used in the main text; the weighted floor is dominated by IMR90, the largest and most variable group (Table S7).

| **Dataset** | **Density full (loops/Mb)** | $v_{0}^{eff}$ **(kb)** | **T (kb)** | **200M** | **100M** | **50M** | **25M** | **10M** | **1M** |
| --- | --- | --- | --- | --- | --- | --- | --- | --- | --- |
| hESC-DSG-DpnII-R1-T1 | 11.49 | 2.8 | 87 | 11.56 ± 0.08 | 11.55 ± 0.07 | 11.59 ± 0.13 | 11.63 ± 0.37 | 11.12 ± 0.55 | 11.48 ± 2.38 |
| hESC-DSG-DpnII-R2-T1 | 10.64 | 2.8 | 94 | 10.67 ± 0.11 | 10.69 ± 0.15 | 10.52 ± 0.34 | 10.59 ± 0.34 | 10.45 ± 0.75 | 10.64 ± 1.66 |
| HFFc6-DdeI-DpnII-R1-T1 | 7.04 | 0.7 | 142 | 7.05 ± 0.02 | 7.05 ± 0.06 | 7.10 ± 0.07 | 7.12 ± 0.15 | 7.08 ± 0.14 | 8.11 ± 1.31 |
| HFFc6-DdeI-DpnII-R3-T1 | 7.94 | 0.7 | 126 | 7.94 ± 0.05 | 7.94 ± 0.04 | 7.93 ± 0.14 | 7.91 ± 0.16 | 7.91 ± 0.32 | 7.52 ± 0.61 |
| hESC-FA-DpnII-R1-T1 | 8.26 | 8.5 | 121 | 8.47 ± 0.39 | 8.29 ± 0.38 | 8.51 ± 0.56 | 8.13 ± 0.85 | 8.67 ± 1.02 | 9.04 ± 2.47 |
| hESC-FA-DpnII-R1-T2 | 8.13 | 9.7 | 123 | 7.89 ± 0.06 | 7.97 ± 0.43 | 7.69 ± 0.76 | 8.03 ± 1.06 | 8.33 ± 1.67 | 8.62 ± 3.07 |
| HFFc6-DSG-DpnII-R1-T1 | 7.35 | 2.2 | 136 | 7.37 ± 0.03 | 7.36 ± 0.06 | 7.41 ± 0.09 | 7.35 ± 0.23 | 7.34 ± 0.58 | 6.79 ± 1.15 |
| HFFc6-DSG-DpnII-R2-T1 | 8.77 | 1.6 | 114 | 8.53 ± 0.60 | 8.54 ± 0.61 | 8.29 ± 0.77 | 8.41 ± 0.82 | 8.30 ± 1.15 | 9.03 ± 1.69 |
| HMEC | 8.62 | 3.1 | 116 | 8.51 ± 0.04 | 8.49 ± 0.06 | 8.44 ± 0.15 | 8.54 ± 0.24 | 8.63 ± 0.37 | 8.65 ± 1.02 |
| IMR90 | 7.35 | 6.4 | 136 | 7.35 ± 0.00 | 7.40 ± 0.34 | 7.30 ± 0.73 | 7.68 ± 0.85 | 7.39 ± 1.50 | 7.38 ± 2.28 |
| KBM7 | 5.68 | 12.7 | 176 | 5.63 ± 0.18 | 5.70 ± 0.19 | 5.56 ± 0.29 | 5.48 ± 0.40 | 5.61 ± 0.66 | 4.82 ± 1.27 |
| K562 | 8 | 4 | 125 | 8.03 ± 0.04 | 8.01 ± 0.05 | 7.95 ± 0.21 | 8.03 ± 0.34 | 7.89 ± 0.56 | 8.14 ± 1.53 |

**Table S1.** Loop density at full depth and at each subsampling depth (mean ± s.d. across independent subsamples, loops/Mb; n, number of subsamples). $v_{0}^{eff}$ and loop period T shown at full depth. T and $v_{0}^{eff}$ were fitted on a grid with 1 kb and 0.3 kb steps; because the dip position is resolved at 1 kb, small SDs at high depth reflect occasional one-bin shifts of the dip. The IMR90 subsamples at 200M contain 83% of the library and are nearly identical. At 1M, the fit reached the grid edge in 5 of 25 KBM7 subsamples and 1 of 25 hESC-FA-DpnII-R1-T2 subsamples.

| **Sample** | **Loop size full (kb)** | **200M** | **100M** | **50M** | **25M** | **10M** | **1M** |
| --- | --- | --- | --- | --- | --- | --- | --- |
| hESC-DSG-DpnII-R1-T1 | 71 | 70.5 ± 0.6 | 70.4 ± 1.5 | 71.4 ± 1.8 | 71.1 ± 2.7 | 71.2 ± 5.0 | 74.6 ± 10.2 |
| hESC-DSG-DpnII-R2-T1 | 75 | 74.0 ± 0.0 | 74.2 ± 0.4 | 73.8 ± 1.9 | 74.2 ± 3.4 | 75.0 ± 5.8 | 71.9 ± 14.0 |
| HFFc6-DSG-DdeI-DpnII-R1-T1 | 104 | 103.5 ± 0.6 | 103.4 ± 1.1 | 105.0 ± 1.4 | 104.6 ± 2.9 | 104.5 ± 4.2 | 112.6 ± 16.4 |
| HFFc6-DSG-DdeI-DpnII-R3-T1 | 102 | 101.2 ± 1.3 | 101.8 ± 0.8 | 101.9 ± 1.2 | 101.2 ± 3.0 | 101.2 ± 4.7 | 100.1 ± 12.3 |
| hESC-FA-DpnII-R1-T1 | 74 | 73.8 ± 1.3 | 75.2 ± 1.5 | 74.3 ± 4.4 | 73.8 ± 7.6 | 73.9 ± 11.6 | 78.2 ± 22.4 |
| hESC-FA-DpnII-R1-T2 | 82 | 82.0 ± 1.4 | 80.8 ± 3.8 | 81.7 ± 4.1 | 82.4 ± 9.7 | 84.0 ± 9.6 | 86.0 ± 24.5 |
| HFFc6-DSG-DpnII-R1-T1 | 106 | 104.8 ± 1.0 | 105.0 ± 1.6 | 105.1 ± 2.4 | 106.2 ± 2.4 | 105.4 ± 4.4 | 102.9 ± 11.4 |
| HFFc6-DSG-DpnII-R2-T1 | 100 | 99.5 ± 1.0 | 99.0 ± 1.0 | 100.0 ± 2.2 | 99.9 ± 3.8 | 99.6 ± 5.7 | 102.2 ± 17.3 |
| HMEC | 100 | 99.8 ± 1.3 | 98.4 ± 3.6 | 97.9 ± 3.7 | 98.7 ± 7.7 | 101.1 ± 10.9 | 101.8 ± 22.1 |
| IMR90 | 91 | 89.8 ± 0.5 | 90.0 ± 1.9 | 91.2 ± 2.9 | 90.2 ± 4.1 | 88.0 ± 7.6 | 91.7 ± 19.8 |
| KBM7 | 100 | 99.2 ± 5.2 | 99.0 ± 1.6 | 99.1 ± 6.1 | 102.0 ± 11.5 | 99.4 ± 13.7 | 98.8 ± 32.1 |
| K562 | 89 | 88.8 ± 1.7 | 89.4 ± 1.5 | 89.7 ± 2.4 | 88.9 ± 4.7 | 88.7 ± 9.4 | 92.2 ± 17.4 |

**Table S2**. Loop size (peak position, kb) at full depth and at 200M, 100M, 50M, 25M, 10M and 1M reads (mean ± s.d. across independent subsamples). Peak slope varies negligibly with depth (s.d. ≤ 0.02 at all tested depths).

| **Group / sample** | **Replicate type** | **n** | **Density CV (%)** | **Peak size CV (%)** | **Peak slope CV (%)** |
| --- | --- | --- | --- | --- | --- |
| ESC4DN-DSG-DpnII | biological | 2 | 5.47 | 3.87 | 1.2 |
| HFFc6-DSG-DdeI-DpnII | biological | 2 | 8.44 | 1.37 | 3.05 |
| HFFc6-DSG-DpnII | biological | 2 | 12.45 | 4.12 | 6.71 |
| IMR90 | biological | 7 | 25.68 | 23.06 | 9.85 |
| k562 | biological | 2 | 3.31 | 1.57 | 4.99 |
| kbm7 | biological | 4 | 11.82 | 5.48 | 3.07 |
| U54-ESC4DN-DSG-DpnII-R1-T1 | technical | 9 | 0.85 | 1.38 | 0.12 |
| U54-ESC4DN-DSG-DpnII-R2-T1 | technical | 9 | 0.77 | 0.94 | 0.13 |
| U54-HFFc6-DSG-DdeI-DpnII-R1-T1 | technical | 9 | 0.24 | 0.58 | 0.08 |
| U54-HFFc6-DSG-DdeI-DpnII-R3-T1 | technical | 9 | 0.4 | 0.98 | 0.1 |
| U54-HFFc6-DSG-DpnII-R1-T1 | technical | 7 | 1.2 | 0.85 | 0.05 |
| U54-HFFc6-DSG-DpnII-R2-T1 | technical | 8 | 1.12 | 1.42 | 0.08 |
| mean (biological) | biological |  | 11.19 | 6.58 | 4.81 |
| mean (technical) | technical |  | 0.76 | 1.02 | 0.09 |

**Table S3.** Coefficient of variation (%) of loop density, loop size and peak slope within biological and technical replicate groups. Mean biological CV (unweighted across groups): density 11.2%, loop size 6.6%, slope 4.8%; mean technical CV: density 0.8%, loop size 1.0%, slope 0.09%.

| **Depth** | **E1 Pearson (%)** | **Sign agree (%)** | **E1 Spearman (%)** | **Comp. strength (%)** |
| --- | --- | --- | --- | --- |
| 200M | 99.2 ± 0.5 | 97.9 ± 1.0 | 99.2 ± 0.5 | 101.2 ± 0.5 |
| 100M | 97.4 ± 1.9 | 95.9 ± 2.2 | 97.4 ± 1.8 | 103.7 ± 1.1 |
| 50M | 93.1 ± 5.4 | 93.0 ± 4.0 | 93.5 ± 4.8 | 108.0 ± 2.3 |
| 25M | 82.5 ± 13.5 | 87.6 ± 7.8 | 84.7 ± 12.1 | 110.7 ± 2.3 |
| 10M | 57.5 ± 29.3 | 76.0 ± 14.5 | 61.3 ± 29.8 | 102.4 ± 18.3 |
| 1M | 1.9 ± 2.0 | 51.2 ± 0.8 | 2.0 ± 1.9 | 14.4 ± 12.2 |

**Table S4**. Compartment metrics (% of full-depth value, mean ± s.d. across 9 libraries). E1 computed at 100 kb; compartment strength with bins ranked by the E1 of each subsample.

| **Depth** | **Ins. Pearson (%)** | **Boundary F1 (%)** | **Boundary prec. (%)** | **Boundary recall (%)** | **Boundary count (%)** | **Boundary strength**  **(%)** |
| --- | --- | --- | --- | --- | --- | --- |
| 200M | 97.9 ± 1.5 | 86.6 ± 5.8 | 84.4 ± 6.6 | 89.0 ± 5.0 | 105.6 ± 3.1 | 100.9 ± 1.8 |
| 100M | 94.4 ± 3.8 | 77.1 ± 8.1 | 72.1 ± 8.7 | 82.9 ± 7.2 | 115.4 ± 5.6 | 103.8 ± 6.2 |
| 50M | 88.8 ± 6.9 | 67.6 ± 8.6 | 60.5 ± 8.8 | 76.9 ± 8.5 | 127.8 ± 7.6 | 111.4 ± 13.6 |
| 25M | 80.3 ± 10.4 | 58.2 ± 8.4 | 50.0 ± 7.7 | 69.9 ± 9.7 | 140.7 ± 8.4 | 128.0 ± 25.5 |
| 10M | 62.1 ± 11.2 | 46.3 ± 7.5 | 39.6 ± 5.8 | 56.0 ± 10.5 | 141.3 ± 9.6 | 168.6 ± 47.3 |
| 1M | 5.0 ± 28.6 | 0.0 ± 0.0 | 5.0 ± 10.0 | 0.0 ± 0.0 | 0.0 ± 0.0 | 479.2 ± 474.3 |

**Table S5**. Insulation metrics (% of full, mean ± s.d. across 9 libraries; 100 kb window, 10 kb resolution, ±2-bin (20 kb) tolerance). Boundary strength is averaged over called boundaries. At 1M, some metrics are undefined in part of the libraries, and the values are computed over the remaining ones: insulation Pearson over 6 libraries (flat track in the other 3), boundary precision and boundary strength over 4 (no boundaries called in the other 5), and boundary F1 over 2 (zero recall). Boundary strength at 1M is shown for completeness but is not interpretable.

| **Dataset** | **SRR** | **Read pairs** | **Used for** |
| --- | --- | --- | --- |
| hESC-DSG-DpnII-R1-T1 | SRR13601502 | 366221722 | biological and technical |
|  | SRR13601503 | 368025123 | technical |
|  | SRR13601504 | 365362813 | technical |
|  | SRR13601505 | 365284109 | technical |
|  | SRR13601506 | 367503926 | technical |
|  | SRR13601507 | 365505998 | technical |
|  | SRR13601508 | 364280252 | technical |
|  | SRR13601509 | 364189618 | technical |
|  | SRR13601510 | 349902453 | technical |
| hESC-DSG-DpnII-R2-T1 | SRR13601511 | 356532010 | biological and technical |
|  | SRR13601512 | 363556777 | technical |
|  | SRR13601513 | 348045715 | technical |
|  | SRR13601514 | 360473406 | technical |
|  | SRR13601515 | 335358898 | technical |
|  | SRR13601516 | 364188789 | technical |
|  | SRR13601517 | 363810868 | technical |
|  | SRR13601518 | 355370082 | technical |
|  | SRR13601519 | 359579356 | technical |
| HFFc6-DSG-DdeI-DpnII-R1-T1 | SRR13601574 | 361132819 | biological and technical |
|  | SRR13601575 | 372805734 | technical |
|  | SRR13601576 | 358372728 | technical |
|  | SRR13601577 | 360248813 | technical |
|  | SRR13601578 | 371052609 | technical |
|  | SRR13601579 | 375557610 | technical |
|  | SRR13601580 | 364494031 | technical |
|  | SRR13601581 | 374383899 | technical |
|  | SRR13601582 | 370816287 | technical |
| HFFc6-DSG-DdeI-DpnII-R3-T1 | SRR13601583 | 368488723 | biological and technical |
|  | SRR13601584 | 371156903 | technical |
|  | SRR13601585 | 368039226 | technical |
|  | SRR13601586 | 367898351 | technical |
|  | SRR13601587 | 361570753 | technical |
|  | SRR13601588 | 357773000 | technical |
|  | SRR13601589 | 351477544 | technical |
|  | SRR13601590 | 369013337 | technical |
|  | SRR14276401 | 364082226 | technical |
| HFFc6-DSG-DpnII-R1-T1 | SRR13601592 | 366276096 | biological and technical |
|  | SRR13601593 | 372186336 | technical |
|  | SRR13601594 | 372929066 | technical |
|  | SRR13601595 | 369938644 | technical |
|  | SRR13601596 | 369800244 | technical |
|  | SRR13601597 | 362508098 | technical |
|  | SRR13601598 | 365208850 | technical |
| HFFc6-DSG-DpnII-R2-T1 | SRR13601599 | 352711374 | biological and technical |
|  | SRR13601600 | 356806888 | technical |
|  | SRR13601601 | 358879540 | technical |
|  | SRR13601602 | 356711707 | technical |
|  | SRR13601603 | 354063938 | technical |
|  | SRR13601604 | 338129653 | technical |
|  | SRR13601605 | 366276446 | technical |
|  | SRR13601606 | 372188125 | technical |
| IMR90 | SRR1658672 | 252725458 | biological |
|  | SRR1658673 | 276809208 | biological |
|  | SRR1658675 | 117797690 | biological |
|  | SRR1658676 | 240438944 | biological |
|  | SRR1658677 | 289367968 | biological |
|  | SRR1658678 | 331634582 | biological |
|  | SRR1658679 | 147914631 | biological |
| K562 | SRR1658693 | 456757799 | biological |
|  | SRR1658694 | 591854553 | biological |
| KBM7 | SRR1658703 | 136881938 | biological |
|  | SRR1658705 | 179038480 | biological |
|  | SRR1658707 | 294486683 | biological |
|  | SRR1658708 | 388468010 | biological |

**Table S6.** Runs used to estimate the biological and technical floors. Each run within a biological group comes from an independent biological experiment: a separately prepared library for Oksuz et al. (R1, R2, R3) and a separate GEO sample for Rao et al.; where several runs came from one sample, the first listed, which was also the largest by reads, was used. Only biological replicates of at least 100M read pairs were included.

| **metric** | **floor definition** | **floor (%)** | **threshold (M read pairs)** |
| --- | --- | --- | --- |
| loop density | mean | 11.19 | 7.2 |
| loop density | n-1 weighted | 16.86 | 2.2 |
| loop density | median | 10.13 | 9 |
| loop size | mean | 6.58 | 20.2 |
| loop size | n-1 weighted | 12.75 | 4.4 |
| loop size | median | 4 | 41.2 |

**Table S7.** Depth thresholds for loop density and loop size under alternative definitions of the biological floor: unweighted mean (used in the main text), n−1 weighted mean and
